# Pharmacokinetics and bioequivalence of sunitinib and Sutent^®^ in Chinese healthy subjects: an open-label, randomized, crossover study

**DOI:** 10.3389/fphar.2023.1294688

**Published:** 2023-11-10

**Authors:** Yanli Wang, Qiaohuan Deng, Zhenyue Gao, Guangwen Liu, Zhengjie Su, Yicheng Zhao, Lixiu Zhang, Haimiao Yang

**Affiliations:** ^1^ The Affiliated Hospital of Changchun University of Traditional Chinese Medicine, Changchun, Jilin, China; ^2^ Chia Tai Tianqing Pharmaceutical Group Co., Ltd., Nanjing, Jiangsu, China; ^3^ Changchun University of Chinese Medicine, Changchun, Jilin, China

**Keywords:** bioequivalence, pharmacokinetics, renal cell carcinoma, sunitinib, safety

## Abstract

**Purpose:** The purpose of this study was to examine the pharmacokinetics (PK), bioequivalence and safety of generic sunitinib and its original product Sutent^®^ in healthy Chinese subjects through a phase-I clinical trial.

**Methods:** The study selected two groups of 24 healthy Chinese subjects in a 1:1 ratio through random allocation. Each participant received either 12.5 mg of sunitinib or Sutent^®^ per cycle. A total of 15 different time points were employed for blood sample collection during each cycle. Furthermore, a comprehensive assessment of the drugs’ safety was consistently maintained throughout the trial.

**Results:** The average adjusted geometric mean ratios (GMR) (90% CI) for the primary PK parameters C_max_, AUC_0-t_ and AUC_0-∞_ were 97.04% (93.06%–101.19%), 98.45% (93.27%–103.91%) and 98.22% (93.15%–103.56%), respectively. The adjusted GMRs for essential pharmacokinetic (PK) parameters all met the requirements for bioequivalence, with values within the acceptable range of 80%–125%. In addition, the two drugs showed comparable results for the other PK parameters. These results indicate that the two drugs were bioequivalent. Furthermore, both drugs showed well safety.

**Conclusion:** The research results proved that the PK and safety profiles of sunitinib in healthy Chinese subjects were comparable to those of Sutent^®^. These results advocate the clinical application of generic sunitinib as a potential alternative to original product Sutent^®^ in the treatment of certain medical conditions.

## 1 Introduction

Renal cell carcinoma (RCC), which develops from proximal tubules of kidney, is regarded as one of the most lethal tumors affecting the urogenital system (Rini et al., 2009). Approximately 15% of patients with RCC will progress to metastatic RCC, greatly reducing their 5-year survival rate to less than 10% (Motzer et al., 1996; Zini et al., 2009). Traditional treatment methods, including surgical resection, radiation therapy, and chemotherapy, are not effective in treating metastatic RCC, and they have numerous side effects (Motzer et al., 1999). As a result, there is an urgent need for more effective treatments for this condition. To overcome the limitations of traditional treatment methods, an increasing number of studies have focused on targeted therapy.

Transmembrane proteins known as receptor tyrosine kinases (RTKs) are important in the signaling and communication between cells (Hicklin and Ellis, 2005). RTKs are widely expressed in various cancer cell types and are known to regulate key cellular processes such as growth, differentiation, and angiogenesis, including metastatic RCC (Östman, 2004). Studies have demonstrated that abnormal activation of certain RTKs, such as Vascular Endothelial Growth Factor Receptor (VEGFRs) and Platelet-derived growth factor receptor (PDGFRs), can stimulate the growth of malignant cells and the development of new blood vessels needed for tumor progression and maintenance (Melnikova and Golden, 2004). By inhibiting the activity of RTKs, targeted therapy drugs can block the signaling pathways that promote tumor growth and metastasis and induce tumor cell death (Butti et al., 2018). Over the past few years, there has been increasing recognition of the significance of RTKs as cancer treatments (Jain, 2005). In particular, Tyrosine kinase inhibitors (TKIs) have shown significant promise in improving the progression-free survival (PFS) and overall survival (OS) of patients suffering from metastatic RCC (Albiges et al., 2012). A study showed that targeted therapies could extend PFS to 27 months and OS to 40 months. (Escudier et al., 2009).

Sunitinib is a multitarget inhibitor of receptor tyrosine kinases that can be administered orally. It is the first targeted drug that can selectively target multiple tyrosine kinase receptors (Abrams et al., 2003; Mendel et al., 2003; O’Farrell et al., 2003; O'Farrell et al., 2003). Sunitinib is approved as a treatment for advanced or metastatic RCC and GIST patients who have either progressed on or are intolerant to imatinib (Papaetis et al., 2008). Sunitinib is effective in producing antitumor effects by blocking the blood and nutrient supply needed for tumor cell growth through the activity of various receptors, including vascular endothelial growth factor. Clinical studies have shown that sunitinib has antitumor activity in a variety of advanced solid tumors (Demetri et al., 2005; Miller et al., 2005; Motzer et al., 2005).

Bioequivalence studies are a method employed to compare the similarity in bioavailability and pharmacokinetics between various drug formulations (Chen et al., 2001). The primary objective of these studies is to ensure that novel formulations do not exhibit significant variations in bioavailability and pharmacokinetics in comparison to reference formulations that have already been approved (Chow and Liu, 2008). These investigations play a crucial role in ensuring drug safety and efficacy, promoting drug innovation, supporting quality management and standardization in the pharmaceutical industry, and providing scientific substantiation for drug registration and approval (Chow and Liu, 2008). The aim of this randomized, open-label, two-cross bioequivalence clinical trial is to explore the PK equivalence of sunitinib, a generic version of Sutent^®^ developed by Chia Tai Tianqing Pharmaceutical Group Co., Ltd. (CTTQ) and Sutent^®^ produced by Pfizer.

## 2 Methods

### 2.1 Study materials

The test formulation, sunitinib malate capsules, was supplied by CTTQ (Bath NO.: 160810132, 12.5 mg), while the reference formulation, Sutent^®^, was provided by Pfizer Inc (Bath NO.: 358EA, 12.5 mg). All study drugs were offered by CTTQ.

### 2.2 Study design

This clinical trial was carried out at the Affiliated Hospital of Changchun University of Traditional Chinese Medicine, Clinical Trial Center (registered number: NCT05800106). The study protocol and its amendments met the Good Clinical Practice guidelines and Declaration of Helsinki. The Affiliated Hospital of Changchun University of Traditional Chinese Medicine Ethics Committee reviewed the protocol and gave final approval for the trial to proceed (approval number: CCZYFYLL 2018-085). Prior to enrollment, the participants received detailed information about the objective of the study, study methods, potential benefits and risks, and possible side effects associated with the drugs. All participants willingly consented to take part in this study and provided written informed consent.

The clinical trial recruited Chinese individuals who were in good health and aged between 18 and 65 years, and the BMI range was 18–28 kg/m^2^. Male subjects had a minimum weight of 50 kg, while female subjects had a minimum weight of 45 kg. The participants underwent a comprehensive evaluation. Subjects who satisfied the eligibility criteria were included, whereas those who fulfilled any of the exclusion criteria were not recruited. Additional details regarding the criteria for including and excluding individuals from the study can be found in the Supplementary Material.

Two groups of subjects were formed in a 1:1 ratio, with one group administered the test drug and the other group receiving the reference drug. On the first day of each dosing cycle, participants, after an overnight fast of at least 10 h, initiate the consumption of a high-fat meal (800–1000 calories) 30 min prior to medication administration. Subsequently, participants orally take 12.5 mg of Sutent^®^ or sunitinib according to the schedule. The washout period between periods was set to be no less than 28 days.

### 2.3 Sample size

To compare the bioequivalence of sunitinib and Sultan^®^, a single-center, randomized, open-label, single-dose, four-cycle study was conducted. Based on previous relevant clinical trials, the coefficient of variation (CV%) for sunitinib C_max_ ranged from 15% to 25% and the CV% for AUC ranged from 5% to 7% (europa, 2020; Tigecycline et al., 2018; Bello and e t al., 2006). We established a β value of 20% (1 - β = 80%), an α value of 0.05, and a θ value of 0.95–1.05, resulting in a final sample size of 24 subjects.

To assess the bioequivalence between sunitinib and Sutent^®^, a single-center, randomized, open-label, single-dose study spanning four cycles was meticulously executed. The anticipated coefficient of variation (CV%) for sunitinib’s maximum concentration (C_max_) ranged within the confines of 15%–25%, while the CV% for the area under the curve (AUC) was projected to be between 5% and 7%. These expectations were grounded in insights gleaned from prior, pertinent clinical trials (europa, 2020; Tigecycline et al., 2018; Bello and e t al., 2006). A robust power analysis was meticulously undertaken, guided by a β value of 20% (equivalent to a statistical power of 80%), an α value of 0.05, and an envisaged θ value spanning the range of 0.95–1.05. This rigorous analysis yielded a definitive sample size of 24 participants, ensuring the robustness of the experimental design.

### 2.4 PK analysis

Blood samples for PK analysis were obtained from the subjects at 15 time points: within an hour prior to drug administration and at 2, 4, 6, 8, 10, 12, 14, 16, 24, 48, 72, 96, 120, and 168 h after dosing. Blood samples were collected at each time point using K2-EDTA anticoagulant tubes, with 3 mL of blood being collected from each subject. The tubes were then promptly placed in ice water to maintain sample integrity. The blood samples were subsequently subjected to centrifugation at 3500 rpm/min for 10 min at a temperature range of 2°C–8°C, and the obtained plasma was preserved at −70°C in a low-temperature freezer for subsequent analysis. The plasma concentrations of sunitinib were analyzed using a well-established liquid chromatography-tandem mass spectrometry (LC-MS/MS) method. The plasma concentrations of sunitinib were determined using a validated liquid chromatography-tandem mass spectrometry (LC-MS/MS) method. The linear range extended from 0.2 ng/mL to 50 ng/mL, and the lower limit of quantification was 0.2 ng/mL, with extraction recoveries ranging from 94% to 95%. Both the intra-batch and inter-batch precision (%CV) fell within the range of 1.9–2.5, and accuracy (RSD) fell within the range of −0.8-1.3.

### 2.5 Safety analysis

During the trial, adverse events (AEs) were recorded, along with clinical observations and vital signs. Any deviations from baseline that were considered clinically relevant were documented as adverse events (AEs). During the study period, safety laboratory tests were conducted from the time of drug administration until 24 h after the final blood sample collection. The clinical researchers continuously monitored and graded the severity of AEs. All recorded AEs were followed up until they were resolved or stabilized.

### 2.6 Statistical analysis

The Phoenix WinNonlin software (Pharsight Corporation, version 6.4 or higher) was utilized to analyze plasma drug concentrations and calculate key pharmacokinetic parameters. The SAS (version 9.4) was used to statistical analysis pharmacokinetic parameters AUC, C_max_, and T_max_. AUC and C_max_ underwent variance analysis after logarithmic transformation, considering four factors: individual, formulation, period, and sequence. T_max_ was analyzed using non-parametric testing. Descriptive statistics, including N (sample size), Mean (average), SD (standard deviation), median, Min (minimum), Max (maximum), %CV (coefficient of variation), and Geomean (geometric mean), were used to analyze PK parameters associated with the administered formulation. Furthermore, quantitative data, such as means, SD, medians, minimum values, and maximum values, were used for the analysis of safety observation outcomes.

## 3 Results

### 3.1 Summary of participant characteristics at baseline

**FIGURE 1:**
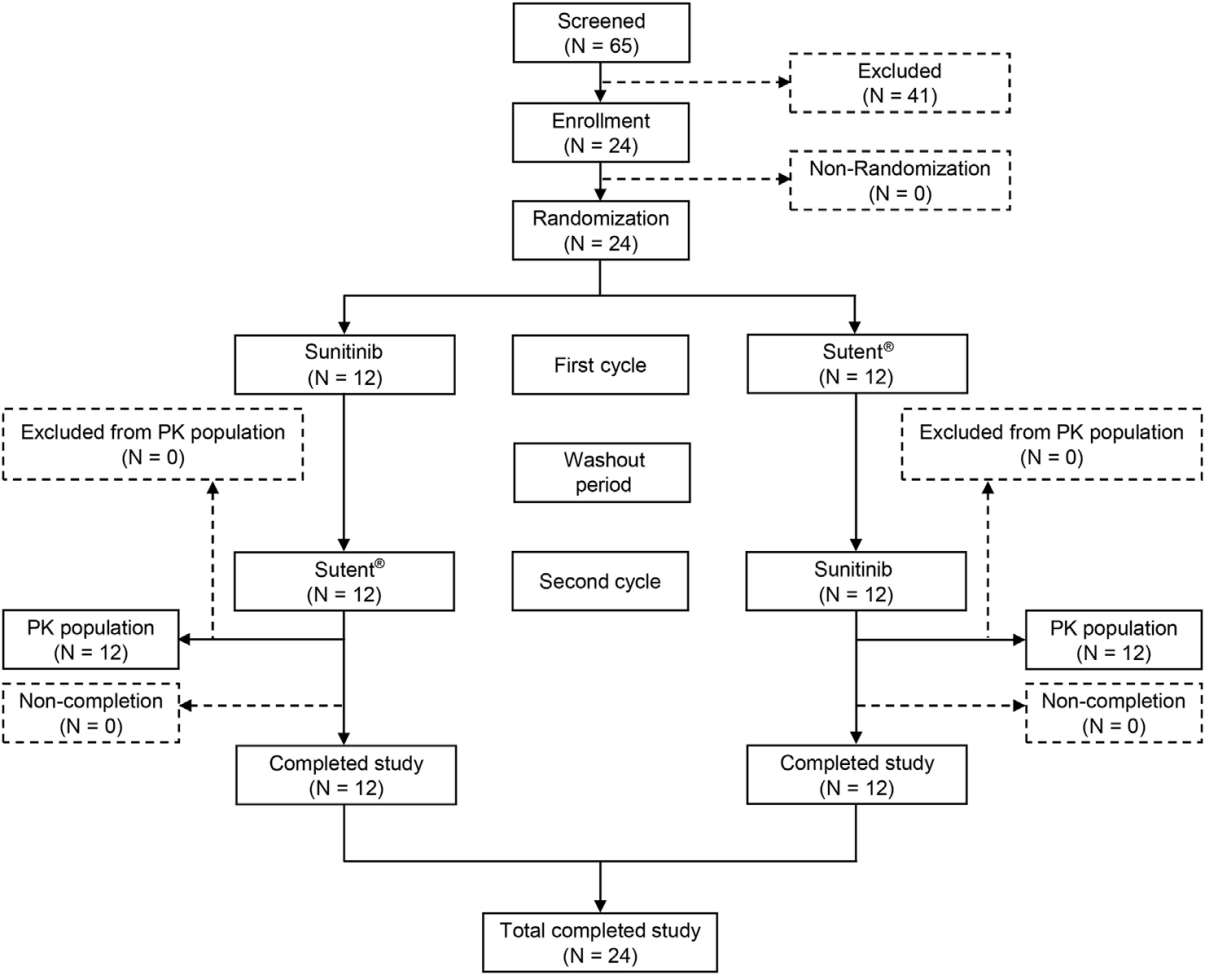
Patient flow chart. PK: pharmacokinetic, n: the number of subjects.

Sixty-five volunteers underwent screening, and 41 were excluded based on exclusion criteria. Finally, 24 male volunteers were enrolled in the trial (Figure 1). Table 1 displays comprehensive demographic data pertaining to the volunteers. The average age of the volunteers was 32.2 ± 6.82 years, the average height was 173.5 ± 6.6 cm, the average weight was 71.1 ± 7.8 kg, and the average body mass index (BMI) was 23.7 ± 2.5 kg/m^2^. All enrolled participants met the inclusion and exclusion criteria without any violations.

**TABLE 1:** Demographic baseline.

Characteristic	N = 24
**Age (Years)**	
N (N miss)	24 (0)
Mean ± Std	32.2 ± 6.82
Median (Q1, Q3)	32.5 (26.5–37.5)
Min-Max	20–44
**Gender**	
Male	24 (100.00)
Female	0 (0.00)
Total	24 (100.00)
**Weight (kg)**	
N (N miss)	24 (0)
Mean ± Std	71.08 ± 7.807
Median (Q1, Q3)	70.60 (66.85–77.90)
Min - Max	51.9–81.1
**Height (cm)**	
N (N miss)	24 (0)
Mean ± Std	173.46 ± 6.597
Median (Q1, Q3)	173.50 (170.75–177.25)
Min - Max	159.5–186.0
**BMI (kg/m** ^**2**^ **)**	
N (N miss)	24 (0)
Mean ± Std	23.65 ± 2.529
Median (Q1, Q3)	23.65 (22.30–25.60)
Min - Max	18.7–27.3

N, number of subjects; SD, standard deviation; BMI, body mass index.

### 3.2 Pharmacokinetic analysis data

**FIGURE 2:**
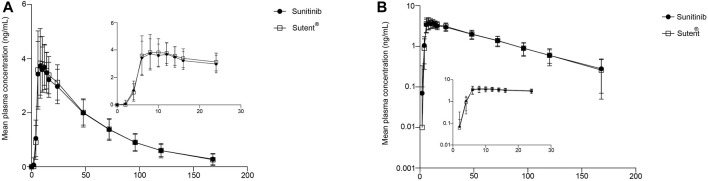
PK analysis of the test drug and reference drug. Mean plasma concentration (±SD) time curve after administration of the test drug and reference drug: arithmetic mean **(A)** and log transformation **(B)**.

A total of 15 time points were sampled for each subject during each cycle, and the plasma concentration of sunitinib was analyzed and collected. The plasma concentration-time curve is depicted in Figure 2A, and the logarithmic transformation of the curve is presented in Figure 2B. The results showed no significant difference in the plasma concentration curves between sunitinib and Sutent^®^ under postprandial condition.

The adjusted average geometric mean ratios (90% CI) for the primary PK parameters C_max_, AUC_0-t_, and AUC_0-∞_ were 97.04% (93.06%–101.19%), 98.45% (93.27%–103.91%), 93.15%–103.56%), respectively. The mean values of C_max_ for sunitinib and Sutent^®^ were 4.21 ng/mL and 4.35 ng/mL, respectively. The mean values of AUC_0-t_ for sunitinib and Sutent^®^ were 228.03 ng*h/mL and 231.49 ng*h/mL, respectively. The mean values of AUC_0-∞_ for sunitinib and Sutent^®^ were 251.98 ng*h/mL and 255.89 ng*h/mL, respectively (Tables 2, 3).

**TABLE 2:** Summary of PK parameters.

PK parameters (units)^a^	Mean ± SD (CV%) (N = 24)				*p*
	n	Test drug	n	Reference drug	
C_max_ (ng/mL)	24	4.21 ± 0.80 (19.14%)	24	4.35 ± 0.91 (20.90%)	0.53
AUC_0-t_ (ng^a^h/mL)	24	228.03 ± 58.60 (25.70%)	24	231.49 ± 56.59 (24.45%)	0.68
AUC_0-∞_ (ng^a^h/mL)	24	251.98 ± 70.42 (27.95%)	24	255.89 ± 66.13 (25.85%)	0.43
T_max_ (h)^b^	24	8.000 (6.00,14.00)	24	8.000 (6.00,16.00)	
%AUC_ex_	24	9.04 ± 2.93 (32.40%)	24	9.26 ± 2.71(29.28%)	
λ_z_ (1/h)	24	0.02 ± 0.004 (23.96%)	24	0.02 ± 0.004 (22.41%)	
t_1/2_ (h)	24	43.30 ± 9.79 (22.62%)	24	43.71 ± 9.55 (21.84%)	
V_d_/F (L)	24	3186.07 ± 617.29 (19.37%)	24	3178.81 ± 682.78 (21.48%)	
CL/F (L/h)	24	53.06 ± 13.25 (24.98%)	24	52.12 ± 13.72 (26.32%)	

^a^ Correct data.

^b^ T_max_ is described by median (min, max).

C_max_: the maximum observed drug concentration in the plasma; AUC_0-t_: the AUC, of the analyte in the plasma over the time interval from time zero to the last measurable concentration; AUC_0-∞_: the area under the curve from 0 to infinity; T_max_: the time from administration to the maximum observed concentration of the analyte in the plasma; AUC_ex_ (%): ((AUC_0-∞_-AUC_0-t_)/AUC_0-∞_) × 100%; λ_z_: terminal rate constant in the plasma; t_1/2_: the terminal half-life of the analyte in the plasma; V_d_ (L): apparent volume of distribution; CL (L/h): the apparent clearance of the analyte in the plasma after extravascular administration.

**TABLE 3:** Bioequivalence statistics of pharmacokinetic parameters.

PK parameters	GLS mean		GMR (%)	Ratio 90%CI (%)	CVw	Power (%)
	T	R				
C_max_ (ng/mL)	4.1312	4.2570	97.04	(93.06,101.19)	8.46	>99.99
AUC_0-t_ (ng*h/mL)	221.2356	224.7217	98.45	(93.27,103.91)	10.93	>99.99
AUC_0-∞_ (ng*h/mL)	243.3397	247.7609	98.22	(93.15,103.56)	10.72	>99.99

PK, pharmacokinetic; CI, confidence interval; GLS, Mean: geometric least square means; GMR, geometric mean ratio; C_max,_ the maximum observed drug concentration in the plasma; AUC_0-t_: the AUC, of the analyte in the plasma over the time interval from time zero to the last measurable concentration; AUC_0-∞_: the area under the curve from 0 to infinity; CVw: CVw, for differences between the test and reference products.

All primary PK parameters had 90% CIs within the range of 80.00%–125.00%, meeting the criteria for bioequivalence. Other PK parameters in Table 2 further support the comparability of the PK profiles of sunitinib and Sutent^®^, thus confirming their bioequivalence.

### 3.3 Safety results

During the trial, all participants remained in good overall health with stable vital signs, and no significant adverse reactions or serious adverse events were reported. Among the 24 participants who completed the study, 7 participants experienced a total of 10 adverse events (Table 4). The adverse events possibly related to the drug, as judged by the investigators, were increased bilirubin, increased blood glucose, increased AST, microscopic hematuria, complete right bundle-branch block, frequent ventricular premature contractions, and frequent ectopic beats. All adverse reactions were resolved or relieved after the end of the trial. These results demonstrate that sunitinib and Sutent ^®^ have good safety profiles in healthy volunteers.

**TABLE 4:** Summary of AEs.

Adverse reactions	Test drug		Reference drug	
	N	Number of subjects (n%)	N	Number of subjects (n%)
**Total adverse events (AEs)**	7	5 (20.8%)	3	3 (12.5%)
**TEAE related to drug**	7	5 (20.8%)	3	3 (12.5%)
Total bilirubin increased	1	1 (4.1%)	0	0
Indirect bilirubin increased	1	1 (4.1%)	0	0
Hyperglycemia	1	1 (4.1%)	1	1 (4.1%)
Glutamyl-transpeptidase Increased	1	1 (4.1%)	0	0
Positive urine occult blood	1	1 (4.1%)	1	1 (4.1%)
Cardiotoxicity	2	1 (4.1%)	1	1 (4.1%)
At least grade 3 AEs	0	0	0	0
SAE	0	0	0	0
Drug-related death	0	0	0	0

TEAE, treatment emergent adverse event; SAE, serious adverse event; Drug-related AEs, were defined as any AEs, that were considered by the investigator to be related to the study drug. n% is the proportion of the number of adverse reactions in all subjects who received sunitinib and Sutent ^®^.

## 4 Discussion

Sunitinib has been approved by regulatory agencies in the United States and Europe for its demonstrated efficacy in extending the survival of individuals diagnosed with metastatic RCC, and gastrointestinal stromal tumors are a potential target for this treatment. This single-center phase-I clinical trial employed a randomized, open-label, crossover design to compare the bioequivalence and safety of sunitinib and Sutent^®^. Previous research on Sutent^®^ has shown a higher variability among patients (Goodman et al., 2007), while PK parameters were similar in healthy individuals and solid tumor patients (Houk et al., 2009). Thus, this study selected healthy subjects as the study population. Population PK analysis showed that age, race and sex had no clinically relevant impact on the *p*Ks of sunitinib (Houk et al., 2009). Consequently, healthy males were chosen as the study’s subjects. Sunitinib undergoes its initial metabolic transformation primarily via cytochrome P450 3A4. This process leads to the formation of its principal active metabolite, SU12662, which is subsequently further metabolized to an inactive form by CYP3A4 (Kassem et al., 2012b). The primary route of elimination for sunitinib is through the feces, accounting for 61% of the total administered dose, whereas renal excretion contributes only 16% (Adams and Leggas, 2007). Sunitinib is unlikely to significantly inhibit or induce CYP enzymes, thus reducing the risk of potential interactions with other drugs or food substances (Zhou and Gallo, 2010; Kassem et al., 2012a). Furthermore, its bioavailability remains unaltered by food consumption (Bello and e t al., 2006). Hence, this study was designed as a postprandial trial. Despite the typical recommendations from the EMA and the FDA to use a 50 mg dose or peak strength for bioequivalence studies (europa, 2020; U.S. Food and Drug Administration, 2022), the existing data indicate that Sutent^®^ exhibits linear PKs, with C_max_ and AUC increasing proportionally with the drug dose (Sakamoto, 2004; Mahmood et al., 2011). Hence, we selected a lower dose of 12.5 mg of sunitinib or Sutent^®^ for this trial. The elimination half-life of sunitinib in healthy subjects’ plasma is approximately 40–60 h; therefore, the washout period of this trial was more than seven times that duration to avoid the effect of the previous cycle. This is sufficient to ensure that at the start of the next dosing cycle, all subjects have drug concentrations below the limit of quantification by bioassay. In this trial, the pre-dose plasma concentrations of sunitinib for each subject were below the quantification limit and showed no carryover effects, indicating the adequacy of the washout period in the trial protocol.

In this study protocol, individual subjects’ PK data will be excluded in the presence of outliers to ensure the accuracy of the analysis. Specific exclusion criteria encompass the following scenarios: 1) The first sample is C_max_, but early post-dosing samples taken within 5–15 min are not collected. 2) Vomiting occurs within twice the time of the median T_max_ in the same group of subjects. 3) Pre-dose blood sample drug concentrations exceed 5% of post-dose C_max_. It is noteworthy that all 24 subjects successfully completed two study cycles without encountering any outliers. As a result, the full analysis set, safety data analysis set, and bioequivalence analysis set consist of these 24 subjects.

During the bioequivalence assessment, primary evaluation indices such as AUC and C_max_ were utilized, with AUC_0-t_ and AUC_0-∞_ being among the parameters examined (U.S. Food and Drug Administration, 2020). In addition, the ratio of PK parameters between the generic drug and the reference drug should have a 90% confidence interval within 80%–125% (Krishnaswami et al., 2015; Li et al., 2021). Additionally, auxiliary evaluation of bioequivalence included several other PK parameters (Miyoshi et al., 2020). The blood drug concentration curves of the two drugs were not significantly different, with the adjusted geometric mean ratios of primary PK parameters C_max_, AUC_0-t_, and AUC_0-∞_ for Sunitinib and Sutent ^®^ meeting the requirements mentioned above at 102.70%, 102.24%, and 102.52%, respectively. Moreover, the secondary PK parameters showed no significant differences between the two drugs, and they fulfilled the required PK criteria for the generic drug. Based on previous study, healthy subjects who consumed a high-fat, high-calorie diet and orally administered 50 mg of sunitinib demonstrated the following pharmacokinetic parameters: C_max_ (ng/mL), AUC_0–t_ (ng*h/mL), and AUC_0-∞_ (ng*h/mL) were 25.1 (21.1–29.7), 1476 (1264–1724), and 1489 (1276–1736), respectively. The T_max_ was 8.03 h (8.0–16.0 h), and the t_1/2_ was 59.1 h (53.4–65.3 h) (Bello and e t al., 2006). These findings support the conclusion of the study and are consistent with previous research.

Individual variability and precision were calculated separately for AUC and C_max_ based on the trial results. The variability rates of primary PK parameters (C_max_, AUC_0-t_, AUC_0-∞_) were 8.46%, 10.93%, and 10.72%, respectively, and the power was >99.0%. These data indicates that the sample size of the trial was sufficient for evaluating the equivalence of Sunitinib and Sutent^®^. The variance analysis of the natural logarithm-transformed pharmacokinetic parameters of sunitinib (C_max_, AUC_0-t_, AUC_0-∞_) indicates that there is no statistically significant impact of sequence, period, and formulation on C_max_, AUC_0-t_, and AUC_0-∞_ (*p* > 0.05) (Supplementary Table S1). The non-parametric test results for T_max_ indicated that the difference between the generic drug Sunitinib and Sutent^®^ in terms of T_max_ does not have significant clinical relevance (*p* = 0.635).

It is important to note that the bioanalytical method used in this study has undergone validation. This method demonstrated excellent linearity, with a linear range from 0.2 ng/mL to 50 ng/mL. The lower limit of quantification was 0.2 ng/mL, and extraction recoveries consistently ranged between 94% and 95%. Both intra-batch and inter-batch precision, expressed as coefficients of variation (%CV), were maintained within a narrow range of 1.9–2.5, indicating high repeatability. The accuracy, measured as relative standard deviation (RSD), was also well-controlled, with values falling within the tight range of −0.8%–1.3%, further affirming the reliability of the analytical method.

Sunitinib has been shown to be effective in the treatment of malignant tumors. However, its use is associated with safety concerns, including cardiovascular toxicity, hematologic toxicity, hepatic toxicity, gastrointestinal toxicity, and other adverse reactions, such as headaches, fatigue, rash, and dizziness (Chu et al., 2007). In this study, 10 mild adverse events were reported during the trial. Fortunately, all of these adverse reactions resolved or improved after the trial ended. It is especially important to monitor patients with a history of coronary artery disease or other cardiac risk factors when using sunitinib. In one case in our study, a subject developed complete right bundle-branch block after taking the reference preparation and frequent ventricular premature after taking the test preparation. Nevertheless, our study demonstrates the good safety profile of sunitinib and Sutent ^®^ in healthy volunteers.

This phase-I clinical trial of generic Sunitinib and Sutent ^®^ in healthy Chinese volunteers confirmed the bioequivalence of the generic drug to the reference drug and produced the expected results. The study’s findings provide valuable evidence for the upcoming stages of clinical trials for sunitinib and Sutent ^®^, as well as promoting the clinical application of domestic generic drugs.

## 5 Conclusion

This bioequivalence study of Sunitinib and Sutent^®^ in healthy Chinese male volunteers demonstrated similar PK and safety profiles. Bioequivalence was established based on evaluation of the main PK parameters. These results support the bioequivalence of the two formulations and demonstrate good safety in healthy subjects.

## Data Availability

The raw data supporting the conclusion of this article will be made available by the authors, without undue reservation.
