# Supplementary material for: Pharmacokinetics and bioequivalence of sunitinib and Sutent® in Chinese healthy subjects: an open-label, randomized, crossover study
Source: Front Pharmacol. 2023 Nov 10;14:1294688. doi: 10.3389/fphar.2023.1294688 (PMC10667676; doi:10.3389/fphar.2023.1294688)
Supplement: Supplementary file 1 [file DataSheet1.docx]

**Pharmacokinetics and bioequivalence of** **sunitinib and** **Sutent^®^ in Chinese healthy subjects: An open-label, randomized, crossover study**

Inclusion Criteria

1. Volunteers signed an informed consent form before the trial and fully understood the trial content, process, and possible adverse reactions;

2. Volunteers were able to complete the study according to the requirements of the trial protocol;

3. Healthy Chinese male adults (including borderline cases) aged 18 to 45 years old;

4. Weight not less than 50 kg and body mass index (BMI) between 18.0 kg/m^2^ and 28.0 kg/m^2^ (including borderline cases, BMI = weight/height^2^);

5. Volunteers have no disease history of heart, liver, kidney, digestive tract, nervous system, mental disorders and metabolic disorders;

6. Volunteers with normal vital signs, physical examination, laboratory tests, electrocardiograms and imaging or no clinically significant abnormalities;

7. Volunteers should ensure appropriate contraception for at least 2 weeks before drug administration and at least 6 months after the last study drug administration (including the volunteer's partner), and ensure the use of one or more contraceptive measures during sexual activity during this period.

Exclusion Criteria

1. Volunteers with a history of neurological, psychiatric, respiratory, cardiovascular, gastrointestinal, hematologic, hepatic, renal, endocrine, musculoskeletal, or other diseases that may affect drug metabolism or safety, as determined by the investigator;

2. Volunteers with a history of swallowing difficulties or any gastrointestinal disease that may affect drug absorption;

3. Volunteers with a history of intracranial bleeding or any disease that increases bleeding risk (such as recurrent epistaxis, purpura, hemorrhoids, acute gastritis, etc.);

4. Volunteers with clinically significant ECG abnormalities or QTc intervals greater than 450 ms;

5. Volunteers with a history of syncope or presyncope;

6. Volunteers with known allergies to sunitinib or its excipients;

7. Volunteers smoked at least 5 cigarettes per day 3 months before screening;

8. Volunteers with a history of drug and/or alcohol abuse (consuming 14 units of alcohol per week: 1 unit = 360mL beer or 45mL of 40% alcohol or 150mL wine);

9. Volunteers who donated blood within 3 months before screening;

10. Volunteers who took any drugs that could change liver enzyme activity 28 days before taking the study drug;

11. Volunteers who have taken any drugs, vitamin products or herbal medicines within 14 days before clinical trial;

12. Volunteers have taken the study drug and participated in other drug clinical trials within 2 months before the clinical trial;

13. Volunteers with abnormal vital sign results;

14. Volunteers with abnormal clinical medical investigation;

15. Volunteers with abnormal chest X-rays;

16. Volunteers with positive results of Hepatitis (including hepatitis B and C), AIDS, and syphilis;

17. Volunteers who screen positive for drugs or have a history of drug abuse in the past five years or have used drugs in the three months prior to the trial;

18. Volunteers who have difficulty collecting blood or who cannot tolerate venipuncture;

19. Volunteers with acute illnesses that occurred during the screening period or prior to study drug administration;

20. Volunteers were unable to comply with ward management regulations;

21. Volunteers cannot complete the trial for personal reasons;

22. Other conditions that the investigator deems unsuitable for inclusion.

Supplementary table. 1. Variance analysis results of Sunitinib PK parameters.

|  | *p* value | | |
| --- | --- | --- | --- |
| Parameters | Sequence | Period | Formulation |
| C_max_(ng/mL) | 0.221 | 0.554 | 0.232 |
| AUC_0-t_ (ng*h/mL) | 0.851 | 0.331 | 0.624 |
| AUC_0-inf_ (ng*h/mL) | 0.840 | 0.384 | 0.566 |
